# Focus on the blind spots of clinician-patient interactions: A critical narrative review of collusion in medical setting

**DOI:** 10.1177/13591053241284197

**Published:** 2024-09-29

**Authors:** Sophia Deliyanidis, Friedrich Carl Stiefel, Céline Bourquin, Laurent Michaud

**Affiliations:** Lausanne University Hospital and University of Lausanne, Switzerland

**Keywords:** clinician-patient interaction, collusion, communication, doctor patient relationship, non-disclosure of medical information

## Abstract

Collusions, interpersonal phenomena with an impact on patients, significant others, clinicians, and care, are mainly described in the psychotherapeutic literature but also occur in the medical setting. Comprehended as an unconscious bond between two or more persons from a psychotherapeutic perspective, definitions and collusive situations described in the medical setting vary. The question arises whether medical collusions, compared to collusions occurring in the psychiatric setting emerge in different clinical situations or are not identified as transference-countertransference experiences, since there is less sensitivity for the unconscious dimensions of care. We systematically reviewed the medical literature on collusions. Even though a read threat, avoidance of unpleasant feelings (mainly anxiety), runs through the described collusions, the unconscious dimensions and associated defensive maneuvers are rarely evoked. Given the expressed desire to act on collusions in medicine, involving third-party psychiatric liaison clinicians, who supervise clinicians, and hereby help to disentangle collusions, could be beneficial.

## Introduction

Commonly, the word collusion refers to an agreement between two or more people who act secretly or illegally to deceive or cheat someone (Ayres, 1987). The concept of collusion is used in the psychoanalytic (Cassorla, 2018), psychotherapeutic (Stiefel et al., 2023a) and group therapy (Stiefel et al., 2023b), as well as medical setting (Ogden and Quirke-McFarlane, 2023). While in the psychotherapeutic setting collusions are understood as transference-countertransference experiences (Gabbard, 2020), definitions and collusive situations described in the medical literature vary (Chaturvedi et al., 2009).

Our clinical and supervisory activities as liaison psychiatrists, research interest in collusion (Deliyanidis et al., 2023; Stiefel et al., 2017; Stiefel et al., 2018a; Stiefel et al., 2019), and findings of two reviews on dyadic and polyadic collusions in the psychotherapeutic setting (Stiefel et al., 2023a; Stiefel et al., 2023b), motivated us to read on collusions published in medical journals. We thus started to wonder whether these collusions refer to clinically different phenomena or if they could also be understood as transference-countertransference experiences. Transference denotes dysfunctional and mainly unconscious relational patterns, beliefs and assumptions affecting patients’ perception of and reactions toward persons (including health care professionals), which have their roots in their development and significant life events; countertransference is understood as the same phenomenon experienced by therapists or other clinicians toward patients (Prasko et al., 2022).

Before reviewing the literature, we will introduce the concept of collusion based on a systemic-psychodynamic perspective.

### Collusions: The development of a working definition

Descriptions of collusions date back to early psychoanalytic writings, appearing under different names such as impasse, bastion, or blind spots (Baranger and Baranger, 2008; Nos, 2014; Stiefel et al., 2023a). The term “collusion” was introduced by the psychoanalyst Dicks (1967) to describe couples, who are linked by an unconscious bond, with each partner’s (pathological) behavior being only understandable when considering the relational dynamic. Later, Willi (1977), a couple therapist, popularized collusion with his book “Couples in Collusion”. As Dicks, he used a psychoanalytic (unconscious dynamic) and systemic perspective (partners relationship) and added a classification of collusions based on developmental difficulties involved (e.g. oral, anal-sadistic, oedipal, narcistic collusions).

Our interest in collusion began, when the second author (FS) supervised a palliative care physician, who presented two situations of collusion (Stiefel et al., 2017). We then reviewed the psychotherapeutic literature to identify clinical facets of collusions such as their triggers, associated emotions, and defense mechanisms; this allowed us to develop a collusions classification grid, which can be used to document manifest features of collusion (Stiefel et al., 2019). When we started research, the question arose how to identify and delineate collusion form other unconscious interactions. Based on the two reviews mentioned (Stiefel et al., 2023a); Stiefel et al., 2023b), we develop a working definition of collusion (Stiefel et al., 2023a), based on the following characteristics: collusion is a specific relational dynamic between two or more persons (i), who share an unresolved and unconscious issue (ii), by which they are interlocked in a defensive maneuver (iii). The issue at stake is avoided on an intrapsychic level by externalization (e.g. projective identification, acting out) (iv), and may pertain to control, intimacy, loss, dependency, domination, and so on (Stiefel et al., 2023a).

This working definition was evaluated in a qualitative study, in which we identified collusions between oncologists and patients presented in audiotaped supervisions, and delineated collusion from other countertransference and transference-countertransference experiences (Deliyanidis et al., 2023). In non-collusive transference-countertransference experiences, protagonists do not share a same unresolved issue: for example, an anxious patient’ transferential clinging (unresolved issue related to dependency, separation anxiety) may provoke counter-transference anger, motivated by the therapist’s feelings of being invaded (unresolved issue related to intimacy). Colluders, on the other hand, are interlocked by the same unresolved issue, share the same “blind spot” and “put oil on fire” by externalization, which leads to a vicious cycle entertaining the collusive dynamic and making it a different experience.

However, collusions do also end, by the rupture of the relationship: for example, an oral collusion in which the husband is “fed” by his wife (unresolved issue over orality) ended by a separation, when the woman needed help due to illness and her husband was unable to reverse the dynamic. In this example, which derives from the clinics of the second author (FS), husband and wife had a development characterized by childhood neglect; the unmet needs to be nurtured, however, was resolved by the partners in an opposite way. The needy husband was constantly seeking attention (from his wife, others), while his wife provided him this attention, satisfying needs (of the husband, others) and scarifying own needs. One may also say that the wife experienced nurturing by proxy. This relational dynamic was never questioned or put under tension, until the wife became ill and needed help and assistance, which the husband was unable to provide. Given this new constellation, the wife-patient decided to leave her husband. Collusions can also end by insight, for example by means of supervision or a couple therapy. While in couples, collusion may be at the very origin of the relationship (match), collusions between clinicians and patients are triggered by words, gestures, attitudes, acts, emotions, or specific situations activating the unresolved issue. In symmetrical collusions, the same stance is adopted toward the unresolved issue (e.g. concerning limits by transgressing rules), and in complementary collusions an opposite stance (e.g. one transgresses, the other rigidly insists on rules) (Stiefel et al., 2023a).

While there are differences between natural (couples) and care settings (clinicians and patients do not choose each other), there are also similarities such as past developmental difficulties creating collusive resonance.

Finally, when clinicians are in the dual role of clinicians and researchers, they face many practical, psychological, and ethical challenges (Hay-Smith et al., 2016), and might thus not be exempt from colluding with the clinician-patients under observation.

## Objectives and methods of the review

The objective of this critical narrative review was to obtain a comprehensive perspective on collusions in the medical setting and to answer the question whether they refer to clinically different phenomena or could be understood as transference-countertransference experiences.

As narrative reviews do not follow a predefined search strategy and provide the authors’ perspective, the review is informed by our experiences as a systemic psychiatrist-psychotherapist (SD), psychodynamic-oriented liaison psychiatrists-psychotherapists and supervisors in the somatic setting (FS and LM) and as a social scientist (CB), who investigates collusions. A critical stance led us to question the literature, add new thoughts, and situate and discuss collusions described in medicine.

**Figure 1.**
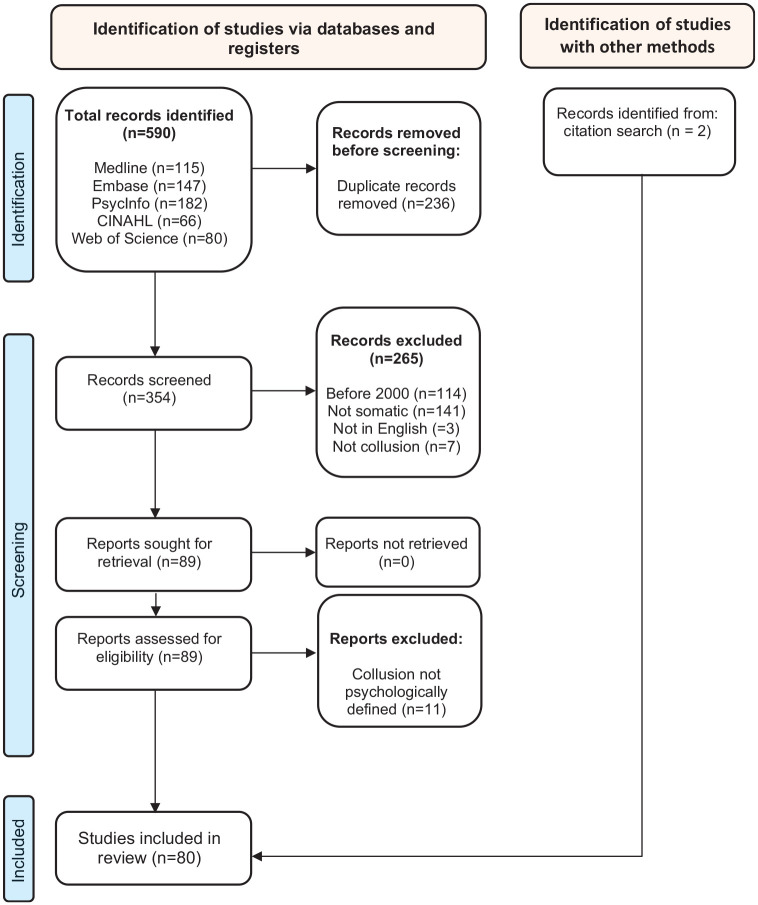
PRISMA 2020 flow diagram for new systematic reviews. Source: Page MJ, McKenzie JE, Bossuyt PM, Boutron I, Hoffmann TC, Mulrow CD, et al. The PRISMA 2020 statement: an updated guideline for reporting systematic reviews. BMJ 2021;372:n71.doi: 10.1136/bmj.n71.

The review was conducted in July 2023, using five bibliographic databases: Medline ALL Ovid, Embase.com, APA PsycInfo Ovid, Web of Science Core Collection, and CINAHL with Full Text EBSCO. Table 1 and Figure 1 provide details regarding search techniques and results, keywords, and index terms used.

**Table 1.** Search strategy.

Medline ALL (Ovid)	Embase.com	APA PsycInfo Ovid	CINAHL with full text (EBSCO)	Web of Science core collection
collusion*.ab,ti,kf. AND (“Professional-Patient Relations”/ OR “Nurse-Patient Relations”/ OR “Physician-Patient Relations”/ OR exp “Patient Care”/ OR exp “Transference, Psychology”/ OR ((patient* ADJ4 relation*) OR (patient* ADJ3 (clinician* OR physician* OR doctor* OR nurse OR nurses)) OR transference* OR countertransference* OR “counter transference” OR “patient* care” OR “therapeutic relation*”).ab,ti,kf.)	collusion*:ab,ti,kw AND (“professional-patient relationship”/de OR “doctor patient relationship”/de OR “nurse patient relationship”/de OR “interpersonal communication”/de OR “patient care”/exp OR “transference”/de OR “counter transference”/de OR ((patient* NEXT/4 relation*) OR (patient* NEAR/3 (clinician* OR physician* OR doctor* OR nurse OR nurses)) OR transference* OR countertransference* OR “counter transference” OR “patient* care” OR “therapeutic relation*”):ab,ti,kw)	collusion*.mp. AND (interpersonal communication/ OR countertransference/ OR ((patient* ADJ4 relation*) OR (patient* ADJ3 (clinician* OR physician* OR doctor* OR nurse OR nurses)) OR transference* OR countertransference* OR “counter transference” OR “patient* care” OR “therapeutic relation*”).mp.)	(TI (collusion*) OR AB (collusion*)) AND (MH “Professional-Patient Relations+” OR MH “Patient Care+ ” OR MH “Transference (Psychology)+ ” OR TI ((patient* N4 relation*) OR OR transference* OR countertransference* OR “counter transference” OR “patient* care” OR “therapeutic relation* ” OR AB ((patient* N4 relation*) OR (patient* N3 (clinician* OR physician* OR doctor* OR nurse OR nurses)) OR transference* OR countertransference* OR “counter transference” OR “patient* care” OR “therapeutic relation* ”)))(patient* N3 (clinician* OR physician* OR doctor* OR nurse OR nurses))	TS=(collusion* AND ((patient* NEXT/4 relation*)(patient* NEAR/3 (clinician* OR physician* OR doctor* OR “nurse” OR “nurses”) OR transference* OR countertransference* OR “counter transference” OR “patient* care” OR “therapeutic relation*”)) OR)
Hits* 115	Hits* 147	Hits* 182	Hits* 66	Hits* 80
*2023-07-10				

One of the authors (SD) screened the literature, including any type of articles (quantitative and qualitative studies, reviews, commentaries, clinical reflection papers, case reports, letters, and congress abstracts) and excluding articles if: they describe collusions outside the somatic field, the term collusion did not appear, were not in English or published before 2000. Of the 89 included articles, 11 addressed collusions with another than psychological meaning (e.g. collusions in a judiciary understanding or to denote lying, which has multiple meanings and origins, does not require participation of at least two protagonists, and refers rather to a moral than psychological category) and were excluded. Two articles were added based on a citation search. This led to 80 publications, of which 20 were empirical studies. The synthesis was produced by the second author (FS) and presented in 6 themes, which were discussed among co-authors: definitions and clinical situations described as collusions; the conscious-unconscious spectrum of collusions; polyadic collusions and contextual determinants; origins invoked for collusions; effects of collusions; and propositions how to handle collusions.

### Ethical considerations

As this is a review study and there were no human participants, we did not seek ethical approval.

## Results

### Definitions and clinical situations described as collusions

A minority of authors do not provide a definition of collusion (Back et al., 2005; Bergqvist and Strang, 2019; Morriss and Gask, 2009; Penson et al., 2001). For example, Back et al. (2005) consider collusion to be one of the pitfalls of communication with cancer patients; the authors specify in a paragraph that collusion occurs when patients hesitate to bring up difficult topics (e.g. prognosis) and physicians do not inform about these topics (“don’t ask, don’t tell” situations). Also, Bergqvist and Stang (2019) observe that “common collusions” between clinicians and breast cancer patients induces that “hope prevails over truth” and prescriptions of late line chemotherapy. Morriss and Gask (2009) describe clinical situations of medically unexplained symptoms (MUS) as collusions, when neither patient nor doctors are satisfied. Finally, Penson et al. (2001) discuss a collusion between a physician-patient and staff complicating different aspects of care, without detailing any further.

Most authors define collusion as a secret agreement (see e.g. a review by Sutar et al., 2022) concerning withholding of medical, most often prognostic, information from patients, significant others, or health care workers in cancer or palliative care, also called “conspiracy of silence” (Gosney, 2007). In general practice, collusions are thought to operate when physicians treat physician-patients and omit to document relevant psychosocial information (Koppe, 2010). In a similar way, practitioners are considered as colluding, when they refer patients who are no longer fit to drive to other physicians (McLoughlin et al., 2017), agree with patients’ unjustified request for sick leave (Carlsen et al., 2020) or avoid psychological suffering of patients with MUS (Morriss and Gask, 2009; Salmon, 2007; Stone and Clarke, 2007). In pediatrics, collusions between clinicians and parents are observed to lead to discharge against medical advice, without addressing the child’s interest (Stanton and Powell, 2013). All these authors remain descriptive, without discussing etiological or pathogenetic aspects.

Finally, a minority of authors rely on the psychotherapeutic definition of collusion. Among them figure Atkinson and McNamara (2017), who describe collusions as unconscious interactions observed in consultations for the prevention of harmful behavior of pregnant women living with obesity: various risk factors, except obesity, are addressed. Ogden and Quirke-McFarlane (2023) attribute a specific type of undermining obese patients’ efforts to lose weight also to collusive, unconscious interactions. Onishi (2021) refers to collusion describing how staff and patients unknowingly collaborate to avoid psychological issues, tied by an unconscious bond. The author provides the example of a patient, who requests assisted suicide, motivated by intense (separation) anxiety, and an oncologist, sharing the same anxiety, who harshly rejects the request instead of empathically exploring it (complementary collusion). Roberts et al. (2023), in a case report, attribute the inadequate discharge of a cancer patient to a polyadic collusion, conceived as an “interconnected response” to an emotional trigger, shared by staff members. Finally, Libow (2002) in her review of factitious disorder by proxy explains the spectrum from a naïve child to passive acceptance and active participation with collusion, based on a concept of Sanders (1995).

#### Commentary on definitions and the semantic field of collusions

Most articles define collusions as non-disclosure of medical information by using euphemism, ambiguous, vague or jargon terms (Krishna et al., 2014). However, some authors consider non-disclosure to be a consequence of the interaction between clinicians and patients, as illustrated by terms such as “shared mind” (Hart, 2022), “permeation of the patient-physician relationship” (Singh et al., 2017), “dissonance between doctor, patient or caregivers” (Sutar et al., 2022), “collaboration” or “clinical activism with patient adherence” (Hasegawa et al., 2022). This is also the understanding of Stone and Clarke (2007), who refer in the clinics of MUS to a shared worry between patients and clinicians to overlook a disease, or by Salmon (2007), who considers collusions over power, dominance, and authority. None of these definitions are right or wrong but definitions should be provided since they orient clinics and research.

Regarding the clinical situations, a red threat runs through these descriptions: collusion is associated with avoidance. Avoidance of breaking bad news, making decisions against patient’s desires (oncology), or addressing emotionally charged (palliative care), potentially offensive (obesity, fit to drive, psychosocial problems) and conflictual issues (MUS, discharge in pediatrics). Avoidance is a consequence of anxiety or other painful feelings; what is not discussed is that the collusive dynamic allows the protagonists to diminish or evacuate these feelings.

### From conscious to unconscious dimensions of collusions

Most authors do not address the question whether collusion has conscious and/or unconscious dimensions. Some consider collusions as decisions, which should be avoided (Gosney, 2007), sometimes invoking moral categories such as honesty (Hart, 2022). Others consider collusions as “inevitable” (Grulke and Bailer, 2010), conveying psychological “needs” (Grulke and Bailer, 2010; Harding et al., 2019; Hart, 2022; Hasegawa et al., 2022; Miller, 2000; Roberts et al., 2023; Singh et al., 2017) or describe them as “necessary” to maintain hope (Miller, 2000). Psychological needs are thought to belong to the patient (Sutar et al., 2022), significant others (Chaturvedi et al., 2009), clinicians (Roberts et al., 2023) or both patients and clinicians (Onishi, 2021).

A minority consider unconscious forces are at work. Stone and Clarke (2007) observe that clinicians’ frustrations and worries may resemble the feelings of their patients with MUS. Others understand collusion as an implicit agreement between clinicians and patients to avoid sensitive topics like prognosis (Morriss and Gask, 2009; Salmon, 2007; Stone and Clarke, 2007). Miller (2000) denounces how patients are enrolled in phase I cancer trials and considers clinicians’ explanations as defensive rationalizations against their uneasiness that these trials serve science and not patients.

As mentioned, a few authors explicitly state that collusions are unconscious clinician-patient interactions (Ogden and Quirke-McFarlane, 2023; Onishi, 2021; Roberts et al., 2023).

#### Commentary on degrees of consciousness of collusion

The spectrum operating in collusions reaches from conscious to inevitable (psychological needs) and unconscious. In a psychotherapeutic lecture, the unconscious does not exclude conscious dimensions; conscious and shared emotional experiences between patients and clinicians (Stone and Clarke, 2007), for example, could be explained by projective identification, a frequently invoked defense mechanism in collusions (Stiefel et al., 2023a). Indeed, colluders—they can be patients, clinicians, partners, or other protagonists of settings in which collusions occur, but here we usually refer to patients and clinicians—often recognize that “something is getting out of hands” or that they do not behave in their habitual way, but the origins and underlying dynamics of collusions remain inaccessible. One could thus say that some dimensions of collusions are consciously experienced by the protagonists; others, corresponding to subconscious dimensions, can be elicited when colluders are invited to describe the situation and associated experiences (e.g. which emotions are felt), and some dimensions remain unconscious and can only be accessed when the unresolved and shared issue can be identified and collusion can be worked through, for example in supervision.

This spectrum may be explained by different degrees of sensitivity to the unconscious dimensions of interactions, with some focusing on the manifest dimensions of collusion such as associated emotions, feelings of uneasiness or deviation from good medical practice (Stiefel et al., 2023a). Different perspectives can co-exist and are worthwhile to investigate. However, when it comes to interventions to identify, prevent, or diminish collusion, a comprehensive approach seems necessary.

### Polyadic collusions and contextual determinants

Polyadic collusions are thought to operate when medical errors or negligence are hidden to preserve clinicians’ or hospitals’ reputation (Cohain, 2012; Lester and Tritter, 2001). Such behavior depends on the individual but also on institutional determinants such as a culture of blame (Cohain, 2012) or physicians’ corporatism and conformism (Lester and Tritter, 2001). Similarly, polyadic collusions are reported among clinicians tolerating inadequate behavior of colleagues (Cohain, 2012); individual inability to assume conflicts might favor such attitudes but institutional determinants also play a role.

Most polyadic collusions are described in oncology and palliative care; this may be explained by the existential threats and intense emotions circulating in these settings. However, other settings such as gynecology (issues related to intimacy) or diabetology (issues related to lack of control) may also produce resonance in patients, clinicians, and significant others.

Organizational determinants of polyadic collusions are addressed by Mitchell et al. (2022), who consider specialization, staff rotation, referrals between specialists and fragmentation of care as leading to a collusive loss of medical responsibility. They inform that their so-called “collusion of immortality” is inspired by Balint’s (2005) “collusion of anonymity,” which describes a similar phenomenon. The same collusions are observed in primary care (Wyatt and Sullivan, 2005) or with psychiatric patients with oral pathologies, with dentists as third party not falling into the trap of collusion (Guerra et al., 2023). Moreover, Forbes et al. (2020) consider that collusive hierarchical behaviors lead to exclusion of nurses in medical decision-making. Finally, collusion between socially desirable patients and enthusiastic carers are thought to lead to overrating satisfaction in questionnaires (McGrath et al., 2005).

An example of multiple collusions embedded in a web of contextual determinants is provided by Kerridge (2017), who observes that unrealistic expectations of cancer patients collude with physicians’ desire to help, an industry aiming to produce, medias running after a story and a society striving for hope.

Finally, cultural determinants are considered to increase prevalence of collusions over withholding of medical information (Sutar et al., 2022).

### Commentary on contexts favoring collusion

Polyadic collusions are formed in a specific context. In medicine, what makes group is the caring mission. However, besides the working task, subconscious forces (basic assumption according to Bion (1969)) operate such as the desire of pairing, which might explain some polyadic collusions. Moreover, the medical institution attracts individuals with a certain valence for caring and repairing, due to own past experiences (Rodenbach et al., 2016), which may lead to what Freud called “furor sanandi” (Jiménez-Avello, 2004) and Balint (2005) the “apostolic function” of physicians, favoring collusions. Parin and Parin-Matthèy (1978) suggested the term “adaptation mechanisms,” in analogy to defense mechanisms, to describe how individual psychopathology matches the roles provided by institutions. Especially institutions harboring contrasting figures tend to favor collusions; for example, in medical settings, the expert-clinician and ignorant-dependant patient (Stiefel et al., 2023b).

Besides clinical setting, the institutional organization may influence occurrence of collusions, as Menzies (1960) observed, who described how fragmentation of care and frequent staff rotation lead to collusions of anonymity (lack of responsibility) with relieving effects for staff, diminishing compassion.

Not mentioned in the literature is the extra-institutional context such as dominant discourses. For example, cancer care is colored by discourses such as the “war on cancer” (Stiefel et al., 2023b) amplifying clinicians’ and patients’ own rhetoric and attitudes, which evolve around the need to fight regardless of advanced disease; instead of introducing palliative care, collusions over the disregard of limits (of life, of curative treatments) are accentuated, leading to therapeutic obstinacy.

While especially invoked for withholding of medical information, we consider that culture is not per se a cause of collusions but may increase their prevalence and shape their expressions. In India, such withholding is frequently observed (Sutar et al., 2022), as in Western countries (Epstein et al., 2016). However, in Western countries information is not completely omitted but often oriented toward therapeutic options avoiding prognostic talk (Helft, 2005), and death is rarely addressed by physicians but triggered by patients’ remarks (Salvadé et al., 2022).

Therefore, a comprehensive view on collusion must consider atmospheric (e.g. attitudes, ways to proceed) and structural (e.g. organization, directives), as well as micro-cultural (e.g. a medical ward) and macro-cultural dimensions (institution, society).

### Origins invoked for collusions

Various origins are invoked for collusive non-disclosure of medical information. Most point toward physicians’ shortcomings such as lack of confidence in communication competences (Chaturvedi et al., 2009; Graham et al., 2005; Lee and Wu, 2002; Victor et al., 2018), stress (Chaturvedi et al., 2009; Kalemkerian, 2005; Low et al., 2009), a desire to protect themselves (Lawton and Carroll, 2005), and fears facing uncertainty (Groopman, 2005) and mortality (Grulke and Bailer, 2010; Harding et al., 2019; Lim et al., 2022; Penson et al., 2001). More favorable explanations refer to clinicians’ need to maintain hope (Chaturvedi et al., 2009; Ellis, 2021; Graham et al., 2005; Harding et al., 2019; Lee and Wu, 2002), to “do the most good” (Schwarz, 2004) and not inflicting harm (Dutta et al., 2020; Kirwan et al., 2003; Lee and Wu, 2002; Mathew et al., 2021; Victor et al., 2018). Therefore, some authors suggest not to use the word “collusion” but “unspoken collaboration” (Graham et al., 2005), less negatively connotated, or add the adjective “necessary” (Helft, 2005).

Collusion over withholding medical information driven by family is almost always understood as a way to preserve hope (Borasio et al., 1998; Graham et al., 2005) or to protect patients from distress (Chaturvedi et al., 2009; Jeba et al., 2016; Seth, 2010; Sutar et al., 2019; Tay et al., 2017; Victor et al., 2018), and rarely to avoid significant others’ own painful emotions (Chaturvedi et al., 2009; Jeba et al., 2016; Vivian, 2006). The family’s collusive intentions are generally qualified as good (Philip et al., 2018), or an expression of love (Lee and Wu, 2002).

Supra-individual explanations for collusions refer to family-centric (Jeba et al., 2016) or paternalistic cultures (Dutta et al., 2020; Krishna et al., 2014; Low et al., 2009; Tay et al., 2017), in contrast to western countries where this is not meant to be the rule (Love and Cook, 2015). However, Harding et al. (2022) argue that families’ pressure in India and Asia might rather be driven by the fear of provoking anxiety in the patient, which is confirmed by local clinicians (Chaturvedi et al., 2009; Dutta et al., 2020; Krishna et al., 2014; Love and Cook, 2015; Low et al., 2009; Radha Krishna and Alsuwaigh, 2015; Tay et al., 2017). Indeed, a prevalent belief (or fear) among families is that disclosure destroys patients’ hope or will to live (Dutta et al., 2020). This belief also operates in Western countries, when clinicians focus on treatment to avoid prognosis (Bergqvist and Strang, 2019) and end of life issues (Back et al., 2005; Bergqvist and Strang, 2019; Chan, 2000). Moreover, individual attitudes of self-surrender in India have also been invoked for such collusions (Sutar et al., 2022), but also Western patients may show no interest in medical results, which then remain unaddressed by physicians (Helft, 2005; Hoff and Hermerén, 2008). Western physicians are also observed to rapidly switch discussions (Davis et al., 2013; Grulke and Bailer, 2010; Hoff and Hermerén, 2008; Singh et al., 2017; The et al., 2001) when the issue of prognosis pops up. This switch labeled “collusive medical activism” is explained by a need of patients and physicians to avoid thinking about the inevitability of death (Lim et al., 2022; The et al., 2001).

This dual perspective is also adopted by Hasegawa et al. (2022), who consider that both patient and oncologist experience a burden in end-of-life discussion, which results in postponing such topics. Similarly, Hart (2022) distinguishes collusively evading truth from actively lying. This view of clinicians and patients “playing together” to avoid unpleasant feelings is shared by Miller (2000) (phase I trials), Kitzinger (2002) (conspiracy of silence surrounding violent couples) or Stone and Clarke (2007) (shared frustration and worries with patients with MUS). Similar collusions have been described in child abuse with social workers colluding with one of the parents (usually mothers), sharing anxiety-induced unawareness of the child's suffering (Joyce, 1997).

#### Commentary on evoked origins, participants, and delineation of collusions

The most frequently mentioned form of collusion, withholding of information, is explained by various clinician-related variables, some of them pointing to deficits and others to empathy. Indeed, confronting patients’ vulnerabilities and finitude may provoke anxieties related to own vulnerabilities and mortality (Armstrong, 1987), which may lead to defensive maneuvers taking the form of verbal tabus. Tabus have social functions; one of them is to diminish anxiety by obscuring some aspects of the threat, it’s naming (Steadman, 1935).

Honorable intentions are assumed for families’ requests of withholding medical information. Besides the fact that intentions may be good and effects harmful, the family is a highly regarded social construct and its toxic aspects have only recently become an object of research (Ogden and Quirke-McFarlane, 2023). To integrate a social perspective on collusions seems therefore necessary.

Finally, cultural determinants may explain prevalence of collusions, but psychological mechanisms are at its origin, and as also demonstrated in India, collusions are associated with patients’ poor coping skills and distress (Sutar et al., 2019).

### Effects of collusions

Withholding medical information is often, but not always, associated with negative effects (Hart, 2022), for example in cases of families who collusively exclude patients from information causing more anguish than comfort (Mathew et al., 2021). Various other negative effects on patients are reported such as hampering treatment seeking, compliance (Sutar et al., 2019) and quality of life (Sutar et al., 2019; Victor et al., 2018), or induction of false hopes (Davis et al., 2013), inadequate optimism (Hasegawa et al., 2022), overestimation of prognosis (Grulke and Bailer, 2010) and poor illness understanding (Payne et al., 2014; Singh et al., 2017). Moreover, collusions seem to hinder patients’ expression of emotions (Crooks, 2001), deviate their attention from important end-of-life issues (Roberts et al., 2023), or cause family debts (Helft, 2005), isolation (Schwarz, 2004) and shorten survival (Costello, 2000).

However, the notions of “necessary collusion,” “natural collusion,” “collusive approach,” “patients’ stated and unstated wishes,” “preservation of hope and provision of time to integrate prognostic information” (Groopman, 2005; Helft, 2005; Singh et al., 2017) point also to positive effects. Ball (2001), a fifth-year medical student, thus raises the question in a letter to the editor, whether it is always best to know, as does Ellis (2021) who considers that collusions maintain hope.

Effects on medical care are also observed. Roberts et al. (2023) and Helft (2005) report that colluding oncologists propose unrealistic treatments. Some authors therefore refer to ethical (Yeung, 2017) and legal (Vivian, 2006) aspects of collusions. Mitchell et al. (2022) found by means of focus groups with clinicians that collusive information withholding can hamper referral to palliative care; this has been confirmed by others (Davis et al., 2013; Harding et al., 2019; Hasegawa et al., 2022). Moreover, related to collusions are delays regarding end-of-life care (Davis et al., 2013; Vivian, 2006), persistent total pain (Mathew et al., 2021), inadequate referrals (Hasegawa et al., 2022), insufficient care plans (Lim et al., 2022), impaired shared decision making (Harding et al., 2022; Schlosser and Desh, 2020), and damage to clinical relationships and practice (Gosney, 2007).

Collusive withholding of information is also thought to have negative impacts on clinicians (Costello, 2000; Schlosser and Desh, 2020; Vivian, 2006) and significant others, deteriorating family dynamics (Borasio et al., 1998; Sutar et al., 2019).

In the context of the capacity to drive, physicians are observed to collusively avoid confronting patients by referring them to another physician. In patients with MUS, colluding physicians seem to avoid psychosocial dimensions and instead prescribe unnecessary investigations (Morriss and Gask, 2009; Salmon, 2007; Stone and Clarke, 2007). Finally, in pediatrics, collusions are observed to lead to discharges against medical advice (Stanton and Powell, 2013). Here, one might guess that both parties “collaborate” in the decision, but for different reasons (clinicians may fear conflict and parents distrust medical care).

#### Commentary on clinical consequences of collusions

Given the negative and positive effects of collusions over withholding medical information, the question “cui bono?” must be raised. Indeed, clinicians generally consider having honorable intentions but the distinction between their own and patient needs is not so easy to make. We have recently illustrated this point regarding positive psychology interventions: good intentions can easily become a prescriptive practice and injunction for patients (e.g. to express feelings and gratitude, show altruism, achieve post-traumatic growth, or serenely say good-by) (Stiefel et al., 2023c).

Regarding collusions in midwifery, general practice, obesity prevention or regarding medical errors, negative effects prevail, except for the referral of the assessment of the ability to drive, which we consider adequate to preserve the alliance.

### Propositions of how to handle collusions

Third party to identify and prevent collusive withholding of information is suggested by Schwarz (2004) and others, who call for the involvement of palliative care specialists (Jeffrey, 2001; Sutar et al., 2022), ethicists (Yeung, 2017), treatment “brokers” (The et al., 2001), mediators (Mathew et al., 2021; Stanton and Powell, 2013) or psycho-oncologists (Sutar et al., 2022). While the role of third parties is often not specified, some authors consider that they should raise awareness regarding collusions (Harding et al., 2022), identify their origins (Roberts et al., 2023) or generate more meaningful understandings (Sutar et al., 2022).

Many authors suggest to improve clinicians’ communication (Back et al., 2005; Groopman, 2005; Hasegawa et al., 2022; Low et al., 2009; Onishi, 2021; Seth, 2010; Singh et al., 2017; Sutar et al., 2019), use checklists (Onishi, 2021), ask specific questions (Singh et al., 2017), provide individualized information, implement biopsychosocial screening tools (Koppe, 2010), maintain hope psychologically (instead by using medical treatments) (Bergqvist and Strang, 2019) and adopt a patient-centered approach (The et al., 2001).

To avoid family-clinician collusions, authors propose a stronger alliance with families (Low et al., 2009; Seth, 2010), increase support (Harding et al., 2022; Low et al., 2009) or raise awareness about collusion (Low et al., 2009). More specifically, Dutta et al. (2020) consider that healthcare professionals should be enabled to explore reasons of collusion, ask relevant questions to elicit patients’ view and facilitate interpersonal communication. Finally, public education programs about patient rights and collusion have also been proposed (Chaturvedi et al., 2009).

#### Commentary on propositions to prevent or diminish collusions

The various propositions of third-party involvement to prevent collusive non-disclosure of medical information illustrate the power of the interactional bond between colluders. The general idea of third party-involvement in this context is, that professionals not directly involved in treatment may contribute with their expertise (in palliative care, ethics, psychology) to identify and disentangle collusions, enable colluders to gain distance and insight regarding the non-medical motivations of their stances and allow them to elicit and discuss their arguments and issues at stake.

Suggestions on how to handle collusions refer to similar approaches. Interestingly, psychiatric liaison clinicians are rarely mentioned as third party, even though one of their main missions is to provide supervision, which is especially precious in situations of collusion (Deliyanidis et al., 2023).

Regarding the recommendation to improve communication, recent guidelines on training indeed call for teaching about the psychology of the patient and clinician, and their interactional dynamics (Stiefel et al., 2018b). Awareness of collusion and support of the family could thus become a topic for training.

## Conclusions

First described in the psychoanalytic literature and since then abundantly discussed for its occurrence in the psychiatric and psychotherapeutic setting, collusion has become an emergent topic in somatic medicine. This evolution is to be applauded, since interactional dynamics of the clinician-patient encounter are known to impact patients, clinicians, their relationship, and medical care. This review critically overviews the different facets of collusions described in the somatic field aiming to encourage medical clinicians but also liaison psychiatrists, other health care professionals such as ethicists, as well as teachers and researchers to become acquainted with this fascinating and clinically most relevant concept. The commentaries inserted in the review reflect on the various thoughts and propositions made in the literature on specific aspects of collusion and address their clinical and scientific implications.
